# AN ASSET-BASED APPROACH TO INCREASE ENGAGEMENT OF OLDER AFRICAN AMERICANS IN HEALTH RESEARCH

**DOI:** 10.1093/geroni/igae098.0890

**Published:** 2024-12-31

**Authors:** Trudy Gaillard, Joan Vaccaro, Donna Neff, Cynthia Morton Padovano, Fern Webb

**Affiliations:** Florida International University, Miami, Florida, United States; Florida International University, Miami, Florida, United States; University of Central Florida, Orlando, Florida, United States; University of Florida, Gainesville, Florida, United States; University of Florida, Gainesville, Florida, United States

## Abstract

Asset-based approach to community engagement research aims to identify community strengths that improve health and health equity. The objective of the current study was to identify strategies expressed by older African Americans (OAA) to increase their engagement in health research. The following report was part of a larger study exploring the impact of intergenerational influence and perceptions regarding willingness to participate in health research among ethnically diverse OAA. Participants were recruited from North, Central, and South Florida using various community engagement strategies. Listening sessions were conducted in dyads; older adult (≥65 years) and a trusted family member/friend (25-64 years). The following results are from OAA (≥65 years). All transcripts were recorded and transcribed verbatim. NVivo software© was used for data management and analysis. There was a total of 14 listening sessions (N= 66). In general, most OAA consulted younger trusted family/friends about health concerns before deciding to participate in research. Key findings concerned, the researcher, goals of the research, and with whom and how information would be shared. Community assets identified that researchers should engage the community by: partnering with local primary care providers (both physicians and nurse practitioners); schools (intergenerational engagement); community liaisons “people who look like me”; and local trusted elected officials. Our results indicate that there are multiple community-based assets that can be used to engage Older African Americans in health research. An asset-based approach with community members identified encouraging suggestions that can be integrated into the design of health research for this population.

